# National Diabetes Month — November 2018

**DOI:** 10.15585/mmwr.mm6743a1

**Published:** 2018-11-02

November is National Diabetes Month. In the United States, approximately 30 million persons are living with diabetes and 84 million with prediabetes (*1*). Persons with prediabetes are at high risk for developing type 2 diabetes, heart disease, and stroke (*2*). Likewise, women who have had gestational diabetes (diabetes during pregnancy) are at increased risk for developing type 2 diabetes later in life (*2*). However, type 2 diabetes can be prevented or delayed through a structured lifestyle change program that promotes weight loss, healthy eating, and increased physical activity (*2*). A report on changes in gestational diabetes in the United States is included in this issue of *MMWR* (*3*).

CDC plays a crucial role in preventing type 2 diabetes and diabetes complications. CDC released an action guide to help pharmacists reach persons at risk for type 2 diabetes who could benefit from the National Diabetes Prevention Program (https://www.cdc.gov/diabetes/prevention). In collaboration with partners, CDC launched “Your Health with Joan Lunden and CDC,” a broadcast miniseries that explores prediabetes and diabetes issues (https://www.cdc.gov/diabetestv), and the first national prediabetes awareness campaign (https://www.DoIHavePrediabetes.org) to encourage persons to learn their prediabetes risk. More information is available at https://www.cdc.gov/diabetes/.
